# German and international studies on SARS-CoV-2 seroprevalence

**DOI:** 10.25646/7024

**Published:** 2020-11-13

**Authors:** Christina Poethko-Müller, Franziska Prütz, Nina Buttmann-Schweiger, Julia Fiebig, Giselle Sarganas, Stefanie Seeling, Roma Thamm, Jan Baumann, Osamah Hamouda, Ruth Offergeld, Lars Schaade, Thomas Lampert, Hannelore Neuhauser

**Affiliations:** 1 Robert Koch Institute, Berlin Department of Epidemiology and Health Monitoring; 2 Robert Koch Institute, Berlin Centre for International Health Protection; 3 Robert Koch Institute, Berlin Department of Infectious Disease Epidemiology; 4 Robert Koch Institute, Berlin Centre for Biological Threats and Special Pathogens, Vice president

**Keywords:** SEROEPIDEMIOLOGICAL STUDIES, ANTIBODIES, SARS-COV-2, SEROPREVALENCE, INFECTIOUS DISEASE EPIDEMIOLOGY

## Abstract

Since the beginning of the year 2020, the SARS-CoV-2 coronavirus has spread globally at a tremendous pace. Studies on the prevalence of SARS-CoV-2 antibodies in the population help estimate the number of people that have already been infected. They also allow an estimate of the number of undetected infections i.e. infections that do not appear in data on officially reported cases. The interpretation of study results needs to consider bias from selective sampling and the diagnostic test properties. To promote networking and co-operation between scientists, the Robert Koch Institute has compiled an overview of the seroepidemiological studies conducted in Germany on its website, which is regularly updated. The RKI conducts searches, for example of press releases, study registry entries or preprint server publications, and contacts the lead investigators of these studies. Of the 40 studies contacted so far, 24 have already provided information (as of 25.06.2020). We can differentiate between studies of the general population, of selected population groups such as healthcare workers, or of ongoing cohorts. This article provides an overview of such studies from Germany, but also of selected international studies. A special focus is set on studies of children and adolescents, which are now of particular interest due to the planned reopening of childcare facilities and schools.

## 1. Introduction

COVID-19 has spread globally at a tremendous pace, and both the disease and the virus that causes it – the novel coronavirus SARS-CoV-2 – are the target of intensive research efforts. Seroepidemiological studies are an important area of research, in particular population-based studies on the prevalence of SARS-CoV-2 antibodies (seroprevalence) in the population or in population groups [1, 2]. Serological studies provide insight into the fraction of people that have already had the infection, including undetected infections (dark figure). This helps assess the demand for healthcare, analyse influencing factors for symptomatic and asymptomatic courses [3], identify particularly affected population groups, and determine the infection mortality rate (lethality). Moreover, the results are highly important to manage infection control measures and evaluate non-pharmacological interventions. The detection of antibodies does not equate to immunity [4, 5] (Info box 1). However, seroprevalence in the population does allow an estimate of how far we are from potential herd immunity.

With its Unity Studies, the World Health Organization (WHO), at an early stage, presented a protocol for conducting population-based SARS-CoV-2 antibody studies, and many studies worldwide are based on this protocol [6]. The primary objectives are to determine seroprevalence by sex and age group, and estimate the fraction of asymptomatic, pre-symptomatic or subclinical infections in the general population. The protocol serves as a framework for selecting study populations, determining the study design and study duration, as well as specimens, and includes a short questionnaire for minimum information from participants. It should be adapted to national conditions, such as availability of resources and laboratory capacities. The protocol has been designed to allow data to be collected quickly and systematically and exchanged in a format that facilitates aggregation, tabulation and analysis worldwide.

**Info box 1:**

**SARS-CoV-2 antibodies**

- Specific IgG-SARS-CoV-2 antibodies indicate a past infection.
- Not all infected persons produce antibodies to the same extent.
- It is still unclear how long antibodies remain detectable [10, 11, 19].

**Immunity**

- Antibodies indicate an immunological reaction to SARS-CoV-2.
- A positive antibody test does not guarantee immunity, a negative antibody test does not exclude immunity [9].

According to current knowledge, the seroprevalence of antibodies at population level is the best indicator of how close a population is to achieving potential herd immunity.

In Germany and internationally, antibody studies are being conducted or are planned, and some analyses have already concluded. This article provides a brief overview of the highly dynamic current state of study with a particular focus on Germany: what are the most important methodological aspects for the evaluation of seroepidemiological studies? What results are already available? Which studies are currently collecting data? In addition, the initial results of international seroepidemiological studies and seroepidemiological studies in children are summarised.

## 2. Methodology

### 2.1 Search strategy

To date, there are only few peer-reviewed publications of seroepidemiological studies on SARS-CoV-2 in the literature databases (PubMed and Embase). This applies in particular to studies from Germany, many of which have begun only very recently. The search was therefore expanded to include manuscripts uploaded to preprint servers (medRxiv, bioRxiv, arXiv, ChemRxiv, preprints.org, ResearchSquare, and Social Science Research Network (SSRN)) prior to formal peer review. The defined search terms in the title of publications were ‘SARS-CoV-2’ AND ‘sero OR antibod OR immune OR immunity OR immunology OR fatality rate OR population-based OR cohort study OR dried blood OR test strategy’. Reports and communications from the WHO, the European Centre for Disease Prevention and Control (ECDC) and the Center for Disease Control and Prevention (CDC) as well as study registries (German Clinical Trials Register, ClinicalTrials.gov) were searched and media coverage was also monitored.

### 2.2 Differentiation of seroepidemiological studies by methodology

The results of seroepidemiological studies can only be evaluated if differences in methodology are taken into account. To assess the influence of bias, information on the sample is particularly relevant, as it allows an estimate of how well the sample represents the analysed population group. The validity of results depends, furthermore, on the type of antibody tests used, laboratory analytical procedures, threshold values for a positive result, as well as the time point and method of blood sample collection. Finally, the transparency of methods and results is decisive for the evaluation of the results [7]. Ideally, this information is included in the study protocol and based on the quality and transparency standards of reporting guidelines for observational studies, of which a version adapted for seroepidemiological studies is also available [8].

**Info box 2:**

**SARS-CoV-2 antibody tests [13, 14, 16, 18-20]**

**Sensitivity** indicates how accurately the test detects individuals with SARS-CoV-2 specific antibodies.

**Specificity** indicates how accurately the test detects individuals who do not have SARS-CoV-2 specific antibodies.

**Overview of sensitivity and specificity of SARS-CoV-2 antibody tests**

For example

- from the Foundation for Innovative New Diagnostics (FIND) [21],
- on the websites of the EU Commission [22],
- from the Federal Institute for Drugs and Medical Devices (BfArM) regarding data on the officially registered tests in Germany [23].

**False positives**

- Proportion depends on the specificity of tests and of pre-test probability (prevalence).
- A high proportion of false positives is possible when the pre-test probability is low (low prevalence).
- Illustrative examples for calculations are available here [13, 20, 24].

**Pre-test probability for a positive result**

- Corresponds to the prevalence in the population when tested without specific cause
- Is higher for testing due to symptoms or contact with an infected person.

The probability of detecting people who have already had the disease varies and depends on the sampling frame, the target population and response rate (participation rate), and does not provide a complete picture of infections in the general population. In particular, samples with voluntary participants and those that achieve a low response rate can produce selection bias. Particularly when comparing groups it is important to assess sampling probabilities of participants, for example in household samples where there are several participants per household.

Regarding the types of antibody tests, a number of methodological aspects need to be taken into account [9]. In seroprevalence studies mainly immunoglobulin G (IgG) antibodies are measured, and to a lesser extent additionally IgA and IgM antibodies. While in most patients with a symptomatic COVID-19 infection viral ribonucleic acid (RNA) can be detected directly by oropharyngeal and nasopharyngeal swabs (in a patient’s throat or nose) a couple of days before the patient develops first symptoms, or within the first week after symptoms appear, IgM and IgG antibody tests become positive only with the beginning of the second week after symptoms first occur [10, 11]. Antibody tests can be semi-quantitative, quantitative (antibody titer) or qualitative. If possible, positive test results should be followed by a second test to confirm the initial result [12]. Generally, these will test for neutralising antibodies (neutralisation test) and use cell cultures to test whether infectious viruses are blocked by antibodies in the serum being tested. The analysis of antibody test results has to take into account in particular the correct administration of tests, sensitivity and specificity, as well as the so-called pre-test probability (prevalence of SARS-CoV-2 infection) [13–16] (Info box 2). Online calculators, illustrative calculation examples and decision trees for the interpretation of test results highlight these correlations, but are not presented in more detail here [13, 17, 18]. In addition to antibody tests, many seroepidemiological studies also include tests for acute infections (direct detection of the virus in a patient’s oropharyngeal and nasopharyngeal swab, generally based on polymerase chain reaction (PCR) tests). Currently, population based estimates combining the total number of those with a positive PCR test and those with a positive antibody test are considered to provide the best available approximation to the total number of persons infected with SARS-CoV-2.

## 3. Seroepidemiological studies from Germany

A number of studies are currently being planned and conducted in Germany to determine SARS-CoV-2 seroprevalence. These studies include both cross-sectional as well as longitudinal studies with study populations that are highly diverse locally and regionally. To gain a more complete picture of overall SARS-CoV-2 infections in Germany, the Robert Koch Institute (RKI) has compiled a regularly updated overview of seroepidemiological studies in Germany on its website (www.rki.de/covid-19-serostudies-germany). The information is provided by the lead investigators of studies who were contacted following the searches described in Chapter 2.1. As of 25 June 2020, the RKI had contacted 40 studies. This article is based on the information already received from 24 of these studies. Information on further studies can be provided to the RKI through a contact form on the RKI website. Detailed study protocols were published for three of the studies on the list; seven studies were registered in the German Clinical Trials Register. At the time of publication, there was one peer reviewed and published scientific report [25] and two preprint papers [26, 27].

The study protocols describe various recruitment procedures: complete surveys of sub-populations, random samples based on population registries, random route household samples, and, also, non-probability convenience samples from that part of the population that is close at hand with diverse recruitment approaches. Semi-quantitative procedures based on enzyme-linked immunosorbent assay (ELISA), quantitative immunofluorescence tests and luciferase immunoprecipitation tests (LIPS assay) were used. Apparently, only one survey so far plans to use a rapid test kit (lateral flow assay). To confirm positive antibody test results some studies use neutralisation assays.

**Figure 1:**
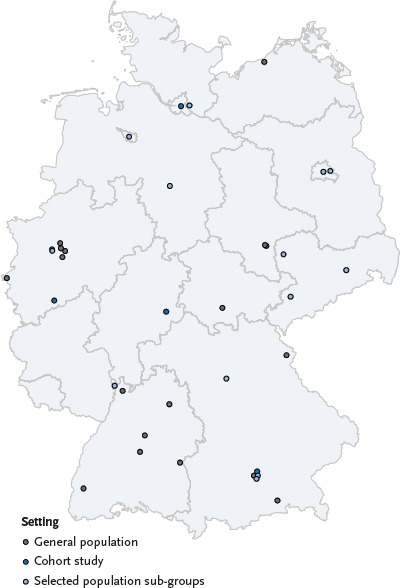
Seroepidemiological studies in Germany, results of the Robert Koch Institute query (as of 25 June 2020) Source: Own diagram

Figure 1 shows a map of Germany indicating where seroepidemiological studies are currently being conducted, based on answers to inquiries in preparation of the RKI website as of 25 June 2020.

### 3.1 Studies of the general population

13 seroepidemiological studies with samples from the general population have so far been identified and presented on the website: three from local hot spots and ten from larger cities or regions.

The Gangelt municipality seroprevalence study conducted by the University of Bonn in the district of Heinsberg, North Rhine-Westphalia, was the first population study to provide results on SARS-CoV-2 seroprevalence for an infection hot spot in Germany. Based on the data from over 900 ELISA IgG (positive or marginal positive) results, complemented by PCR testing, the authors conclude that 15.5% of inhabitants have been infected with SARS-CoV-2. Further key results are that 22.2% of these were asymptomatic infections, as well as that the number of those infected had previously been underestimated (80% of infections had not been officially registered, i.e. the 15.5% seroprevalence was approximately five times higher than prevalence calculated according to the number of registered cases). For early April, an infection fatality rate of 0.36% was calculated [27]. The results of the Gangelt study and of the upcoming hot spot studies are not representative of Germany as a whole.

A further hot spot study was begun in June 2020 in the district of Tirschenreuth, Bavaria (TiKoCo, ‘Seroprävalenz und -inzidenz Studie im Landkreis Tirschenreuth’), and is designed as a repeated cross-sectional survey [28].

The RKI is locally conducting the study ‘CORONA-MONITORING lokal’ [29] in four municipalities that have been particularly affected by the pandemic. Data is being collected through direct tests for a SARS-CoV-2 infection (PCR) as well as through testing for IgG antibodies against the virus validated by neutralisation tests. The aims are to estimate seroprevalence, the proportion of previously undetected cases and the proportion of asymptomatic infections in order to understand the actual distribution of infections in the population. Additional objectives are to determine factors associated with a symptomatic or asymptomatic course, sequelae and the different dynamics of transmission. In May 2020, a study in the Kupferzell municipality (Hohenlohe district) was begun and followed in June by a study in the Bad Feilnbach municipality (Rosenheim district). In each of the four locations, 2,000 randomly selected adults are tested [29].

Moreover, the Helmholtz Centre for Infection Research is also conducting regional hot spot studies. The aim is to determine local levels of immunity in representative samples and to investigate regional differences and time trends. The study began in early July in the Reutlingen district and, over the coming months, will be expanded to include seven further districts [30].

Further studies have been conducted in cities. The prospective COVID-19 cohort Munich study (KoCo19), conducted by the Ludwig Maximilian University (LMU), has completed the first cross-sectional examination of 3,003 randomly selected Munich households. The results are expected for the end of July and the study will be continued longitudinally [31, 32].

Two studies are conducted in Halle (‘COVID-19 Antikörperstatus Halle/Saale’ and ‘Bevölkerungsbasierte Forschungsplattform für COVID-19 Epidemie’), one in Stuttgart (‘Ausbreitung des neuen Coronavirus (SARS-CoV-2) und die gesundheitlichen Folgen’) [33]. The COVID-19 study in Neustadt am Rennsteig, together with a study focused on mothers with children under ten years of age in Rostock [34] and a study of pregnant women in the Franken region co-ordinated at the Friedrich-Alexander Universität Erlangen-Nürnberg [35], represent further studies of the – mainly – adult population at local and regional levels.

Further larger-scale studies have been announced. A Germany-wide seroepidemiological study (‘CORONA-MONITORING bundesweit’) by the RKI together with the German Institute for Economic Research (DIW) plans to use the existing infrastructure of the DIW’s Socio-Economic Panel (SOEP) and conduct questionnaire-based interviews with the 30,000 SOEP participants. In addition, PCR testing and IgG antibody testing is planned with self-administered swabs and kits for blood sampling.

### 3.2 Studies of selected population sub-groups

Rapidly, an increasing number of studies is also being conducted for selected population groups, for example healthcare workers, employees and people living in care homes, or among patients hospitalized for non-COVID-related reasons. Studies of hospital staff in Munich, Reinbek, Hannover and Fulda have been conducted (complete surveys in Munich and Reinbek). A study from Bremen focused on public sector employees.

Blood donor specimens selected by a standardised procedure offer a relatively low-threshold access for seroepidemiological studies. Since April 2020, a study conducted by the RKI in cooperation with blood donation services (SeBluCo) aims to analyse 5,100 blood donor specimens every two weeks from 29 testing regions across Germany to assess the spread of the virus over time. Blood donors represent a good proxy for the healthy general adult population.

Some studies have used established cohorts from longitudinal surveys to gain study participants, such as the Rhineland study [36], the COVID-19 module of the Hamburg City Health Study and the Fr1da-COVID19 study. Such an approach is also possible for the German National Cohort, which has already begun interviewing participants [37]. The detailed information already available for cohort study participants is particularly appropriate to analyse the risk factors for an infection or certain disease courses.

## 4. Seroepidemiological studies in other countries

Seroepidemiological studies have been initiated in many countries, beginning in March 2020 increasing from April in a very short time using the then available antibody tests. A group of researchers from six renowned international universities has compiled and regularly updates and presents on a ‘dashboard’ a summary of international studies. This includes tables that describe the methodologies in detail, important results and links to reports and/or publications [38]. Furthermore, the first 23 studies that were identified (as of 1 May 2020), were critically assessed and summarized in a systematic rapid review published as a preprint article in order to rapidly provide the available evidence. The substantial methodological heterogeneity of these early studies was highlighted, in particular with regard to types of tests, sampling procedures and case numbers [39]. For some studies, only media reports were initially available, and in some cases preliminary results became known before the study design was published. This was criticised in the media, including via social media channels [40].

A more recent compilation of European studies was published in the ECDC Rapid Risk Assessment on 11 June 2020 [41]. Most of the regional studies of the general population or of blood donors show seroprevalences of one-digit [39, 41–43]. Very severely affected regions such as the Ischgl municipality in Tirol and the Italian town of Bergamo showed a considerably higher seroprevalence in the population (42.4% in Ischgl and 57% in Bergamo) [44, 45].

Related to risk factors such as working in the healthcare sector in highly affected regions, seroprevalences of around 33% have been reported for New York City and Bergamo [45, 46]. In more severely affected regions, significant social differences were found [47, 48]. At this point in time, even higher prevalences need critical methodological review, for example when studies in hot spots are conducted with a small number of voluntary participants [49] or when an outbreak at a school is later serologically analysed [50].

From May 2020, large national seroprevalence studies have been announced by several countries, for example by the USA [51], Italy [52], Spain and the United Kingdom [53]. Already available results from Spain for the second wave involving 63,564 participants from 52 regions show an average seroprevalence of 5.2% with important regional differences up to a seroprevalence of 14.7% [54]. As an intermediate result, the United Kingdom has reported a seroprevalence of 5% or higher, and 17% in London [53].

More recent findings indicate that SARS-CoV-2 began to spread outside of Asia as early as January 2020 [55, 56]. This also holds true for some parts of the African continent. The majority of African countries are supposedly in an early stage of the pandemic with increasing case numbers. However, in some countries respiratory infections have increased [57]. Due to the limited public health and surveillance capacities of many countries in the region, an undetected spread of COVID-19 even before April 2020 would seem possible. In this context, determining subnational seroprevalences in African but also South American populations could contribute to developing national and regional COVID-19 containment strategies (for a first study from Brazil, see [58]). Within the framework of existing projects, the RKI currently supports a number of countries, with a focus on the African continent, in preparing and implementing seroepidemiological studies, for example in Malawi and Nigeria.

## 5. Seroepidemiological studies in children

Compared to adults, children with SARS-CoV-2 infection are more frequently asymptomatic or have only mild symptoms. For this reason, acute SARS-CoV-2 infections are less frequently detected through direct virus tests in children [59–62]. Seroepidemiological studies in children are therefore particularly important, because they can determine – without having to rely on the presence of symptoms – whether a person has been infected with SARS-CoV-2. In particular with regard to the reopening of childcare facilities and schools, there is currently a great need for valid data on children and adolescents’ SARS-CoV-2 antibody status, ideally as a trend over time.

In Germany, several seroepidemiological studies based on various study designs are being conducted in children. One such study involving 1- to 10-year-old children is being conducted in Baden-Württemberg (convenience sample). Interim results based on the examination of 2,466 parent-child pairs showed a 0.6% (7/1,120) seroprevalence for 1- to 5-year-old children, a 0.9% (12/1,346) seroprevalence for 6- to 10-year-old children and 1.8% (45/2,466) for parents [63]. Further studies of the general population that include children are conducted, for example in Stuttgart (population registry sample ≥5 years) and the KoCo19 cohort study in Munich (random route household sample). In the Ruhr region, the Corkid study will determine the antibody status of 3,000 children and adolescents aged under 18 during routine medical check-ups [64].

Antibody studies conducted in selected population groups include the CORONA study with children from schools in Leipzig, Dresden and Zwickau, grades one to eight [65]. A time series study conducted in 14 large paediatric clinics from across Germany is recruiting 0- to 18-year-old patients [66]. Tests for SARS-CoV-2 antibodies among children are also being conducted in the already established Fr1da cohort study (2- to 10-year-old children) in Bavaria, a children’s cohort for the early detection of type 1 diabetes [67]. A further study in the form of a cooperation project between the German Youth Institute (DJI) and the RKI has also begun (Corona-KiTa study). The study focuses on children in preschool childcare facilities and aims to examine how transitioning from restricted access to childcare during lockdown to gradual reopening of facilities is related to an increase in infections among children, staff and parents.

Internationally, Switzerland and Sweden have provided initial seroepidemiological results in children. In the canton of Geneva, the population representative SERO-CoV-POP study was conducted, which involved randomly selected participants from the respondents of an annual health survey and their household members (≥5 years). After five of the planned twelve weekly serum surveys (6 April – 9 May 2020), the SARS-CoV-2 seroprevalence for 5- to 9-year-olds (n=123) was 0.8% and 9.6% for 10- to 19-year-olds (n=332). In comparison, the corresponding prevalence for 20- to 49-year-olds (n=1,096) was 9.9% [42]. Interim results from a Swedish study based on residual serum samples (serum samples collected for other analyses) from outpatient care showed seroprevalences of 7.5% for 0- to 19-year-olds, 6.5% for 20- to 64-year-olds and 2.9% for the age group 65 years and older during the survey period between 11 and 17 May 2020. These results are particularly important because, unlike most other countries, Sweden kept the majority of its schools and its childcare facilities open during the pandemic [68].

## 6. Conclusion

In summary, a large number of seroepidemiological studies on SARS-CoV-2 in Germany and internationally have been initiated in order to determine urgent questions concerning the seroprevalence in specific settings, regions and population groups. Due to the considerable psychosocial, economic and societal costs of a general lockdown, there is considerable interest in understanding the regionally differentiated dynamics of infections, also against the backdrop of measures that have already been taken. The initial (preliminary) results thereby consistently highlight that thus far no country has managed to achieve anything close to herd immunity [69].

The criticisms of some of these early studies, as well as the accelerated and, to a certain degree, oversimplified reporting, have created a broader understanding of the possibilities and limits of seroepidemiological studies and the importance of study methodologies. Methodological developments are already visible in the update of the WHO protocol for population-based serological studies [12], as well as the international efforts towards standardisation and reporting [38, 39]. A large number of further regional seroepidemological studies would have to be questioned if they were conducted only in order to answer the question of achieving local herd immunity at this point. Further research questions for seroepidemiological studies therefore appear all the more important, for example concerning the dynamic with which the disease spreads, the extent and patterns of underdetection of infections compared to reported cases, the proportion of asymptomatically infected individuals by age group, the risk and protective factors for an infection, how antibodies develop over time, as well as the long-term health impacts of infections.

While taking into account the heterogeneity of study designs and samples, as well as laboratory analysis procedures, the results from the seroepidemiological studies in numerous regions and settings can increasingly be used for modelling the course of the pandemic [14, 70]. It is important to recognise, at the high pace dictated by the pandemic, when study results apply only locally or in a specific time period and when they can be generalised. The joint effort of different studies and the development of common research questions will be important to gain a better understanding of the pandemic dynamics and to provide an epidemiological database that can help to design effective and proportionate interventions.

## Key statements

- Seroepidemiological studies provide information about the number of people in a population that have already been infected, including previously undetected infections.
- Sampling design and test types can considerably influence the results.
- Over 40 seroepidemiological studies had been started in numerous settings and based on diverse methodologies in Germany by the end of June 2020.
- Data on SARS-CoV-2 infections in children are needed in the context of childcare facilities and schools.
- A more comprehensive picture can be gained from a joint appraisal of current and future studies.
